# Analysis of the relationship between LET, γH2AX foci volume and cell killing effect of carbon ions using high-resolution imaging technology

**DOI:** 10.1093/jrr/rrac098

**Published:** 2023-01-07

**Authors:** Takahiro Oike, Sangeeta Kakoti, Makoto Sakai, Akihiko Matsumura, Tatsuya Ohno, Atsushi Shibata

**Affiliations:** Gunma University Heavy Ion Medical Center, 3-39-22, Showa-machi, Maebashi, Gunma 371-8511, Japan; Department of Radiation Oncology, Gunma University Graduate School of Medicine, 3-39-22, Showa-machi, Maebashi, Gunma 371-8511, Japan; Signal Transduction Program, Gunma University Initiative for Advanced Research (GIAR), Gunma University, 3-39-22, Showa-machi, Maebashi, Gunma 371-8511, Japan; Department of Radiation Oncology, Advanced Centre for Treatment Research & Education in Cancer (ACTREC), Tata Memorial Centre, Homi Bhabha National Institute, Navi Mumbai 410210; Gunma University Heavy Ion Medical Center, 3-39-22, Showa-machi, Maebashi, Gunma 371-8511, Japan; Gunma University Heavy Ion Medical Center, 3-39-22, Showa-machi, Maebashi, Gunma 371-8511, Japan; Gunma University Heavy Ion Medical Center, 3-39-22, Showa-machi, Maebashi, Gunma 371-8511, Japan; Department of Radiation Oncology, Gunma University Graduate School of Medicine, 3-39-22, Showa-machi, Maebashi, Gunma 371-8511, Japan; Signal Transduction Program, Gunma University Initiative for Advanced Research (GIAR), Gunma University, 3-39-22, Showa-machi, Maebashi, Gunma 371-8511, Japan

**Keywords:** carbon ions, linear energy transfer (LET), cell killing effect, γH2AX foci, high resolution imaging

## Abstract

The strong cell killing effect of high linear energy transfer (LET) carbon ions is dependent on lethal DNA damage. Our recent studies suggest that induction of clusters of double-strand breaks (DSBs) in close proximity is one of the potential mechanisms. However, the relationship between LET, the degree of DSB clustering and the cell killing effect of carbon ions remains unclear. Here, we used high-resolution imaging technology to analyze the volume of γH2AX foci induced by monoenergetic carbon ions with a clinically-relevant range of LET (13–100 keV/μm). We obtained data from 3317 γH2AX foci and used a gaussian function to approximate the probability (*p*) that 1 Gy-carbon ions induce γH2AX foci of a given volume (*v_th_*) or greater per nucleus. Cell killing effects were assessed in clonogenic assays. The cell killing effect showed high concordance with *p* at *v_th_* = 0.7 μm^3^ across various LET values; the difference between the two was 4.7% ± 2.2%. This relationship was also true for clinical carbon ion beams harboring a mixed LET profile throughout a spread-out Bragg peak width (30–120 mm), with the difference at *v_th_* = 0.7 μm^3^ being 1.6% ± 1.2% when a Monte Carlo simulation-derived dose-averaged LET was used to calculate *p*. These data indicate that the cell killing effect of carbon ions is predictable by the ability of carbon ions to induce γH2AX foci containing clustered DSBs, which is linked to LET, providing the biological basis for LET modulation in the planning of carbon ion radiotherapy.

## INTRODUCTION

Carbon ion radiotherapy (CIRT) shows promising antitumor effects against a wide spectrum of cancers, including those resistant to conventional photon radiotherapy [1–8]. The cell killing effect of carbon ion beams per unit absorbed dose is linear energy transfer (LET)-dependent up to approximately 100 keV/μm [9–11]. The LET of a carbon ion beam varies along its beam depth, and becomes the highest around the Bragg peak [12]. In a clinical setting, a tumor receives a series of monoenergetic carbon ion beams integrated in the direction of beam depth [13, 14]. Thus, the LET distribution within a given tumor becomes inhomogeneous [15–17]. This indicates that modulation of LET distribution in the treatment planning possibly contributes to improve the outcome of CIRT.

DNA is the principle target for radiation-induced cell killing [18]. Double-strand breaks (DSBs) are considered the most critical among radiation-induced DNA lesions [19]. Using super-resolution microscopy, we recently found that carbon ions induce a greater number of multiple DSBs within a distance of a few hundred nanometers (referred to hereafter as clustered DSBs) than X-rays [20]. Such DSB clustering is also observed by other high LET heavy ion particles [21, 22]. Importantly, this phenomenon is observed not only in cultured cells but also in clinical tumor specimens [12]. In a given clustered DSBs, mis-rejoining of two DSBs induced at distinct chromosomes that lie adjacent to each other can lead to chromosomal rearrangements, including dicentric and translocation; this can cause chromosome mis-segregation during the next mitosis, followed by a type of cell death called mitotic catastrophe [23]. In accordance with this, we demonstrated the superiority of carbon ions over X-rays (per unit absorbed dose) for induction of clustered DSBs at chromosome boundaries [24], as well as post-treatment mitotic catastrophe [25, 26]. Taken all together, the data suggest that the degree of DSB clustering is an important parameter that determines the cell killing effect of carbon ions. However, the relationship between LET, the degree of DSB clustering and cell killing effects remains unclear. Thus, this study aimed at elucidating the relationship between LET, the degree of DSB clustering and cell killing effects of carbon ions using a high-resolution imaging by advanced microscopic technology.

## MATERIALS AND METHODS

### Cell culture and irradiation

The human lung cancer cell line A549 and the human fibroblast line 1BR hTERT were cultured in Eagle’s Minimal Essential Medium (Wako, Osaka, Japan) and Dulbecco’s Modified Eagle’s Medium (Wako), respectively, supplemented with 10% fetal calf serum (Sigma-Aldrich, St. Louis, MO, USA). Carbon ion irradiation was performed at Gunma University Heavy Ion Medical Center (GHMC) [20, 27]. Monoenergetic beams (energy = 290 MeV/n; LET = 13, 20, 40, 60, 80, or 100 keV/μm) or clinical beams (energy = 290 MeV/n; center of the spread-out Bragg peak [SOBP]; SOBP width = 30–120 mm in 10 mm increments) were used. Horizontal and vertical beam directions were used for immunofluorescence staining and clonogenic assays, respectively [20, 25, 26]. For all experiments, 1 Gy was used.

### Immunofluorescence staining

At 30 min post-irradiation, cells seeded on coverslips were fixed with 3% paraformaldehyde (Sigma-Aldrich)–2% sucrose (Sigma-Aldrich) for 10 min at room temperature. The fixed cells were permeabilized for 3 min at room temperature with 0.2% Triton (Sigma-Aldrich) and washed with phosphate buffered saline ([PBS] Sigma-Aldrich). The cells were incubated for 30 min at 37°C with a primary antibody specific for γH2AX (05-636, Millipore, Burlington, MA, USA) prepared in 2% bovine serum albumin ([BSA] Sigma-Aldrich)–PBS. The cells were then incubated for 30 min at 37°C with a secondary antibody conjugated to Alexa-Fluor-488 (Thermo Fisher Scientific, Waltham, MA, USA) prepared at 1:500 in 2% BSA–PBS containing 0.1 mg/mL 4′,6-Diamidino-2-Phenylindole, dihydrochloride (DAPI; Roche, Mannheim, Germany). After washing with PBS, coverslips were mounted on slide glasses (Matsunami, Osaka, Japan) using Vectashield (Vector Laboratories, Burlingame, CA, USA).

### Measurement of γH2AX volume using DeltaVision OMX and Imaris

3D immunofluorescence image with a deconvolution was obtained by a microscope system DeltaVision OMX (version 4, GE Healthcare, Chicago, IL, USA). Images were acquired using a Plan Apo *n* × 60, 1.42 NA oil immersion objective lens (Olympus, Tokyo, Japan) and a liquid-cooled sCMOs camera (PCO, Kelheim, Germany). Z stacks were taken over 3–5 μm areas to cover an entire nucleus (sections taken every 0.25 μm). Deconvolution was performed by softWoRx software. After raw images were obtained, three dimensional γH2AX polygon rendering was performed using surface mode in Imaris 8.1.2 (Bitplane, Zurich, Switzerland), and the γH2AX object volume was measured. Thirty nuclei per LET per cell line were evaluated. γH2AX foci showing less than ~0.01 μm^3^ was excluded for the analysis.

### Analysis of γH2AX signal positivity using conventional fluorescence microscopy

Images of immunofluorescence-stained samples (prepared as described in the previous section) were taken using an ECLIPSE Ni-U with DS-Qi2 camera (Nikon, Tokyo, Japan) with a 40× lens, and analyzed using NIS-Elements D software (Nikon). For each experimental setting, at least 200 cells within four to five fields were recorded. The γH2AX and DAPI signals were identified by ImageJ (version 1.48, National Institutes of Health, Bethesda, MD, USA), from which intranuclear occupancy by γH2AX signals was calculated. Unhit cell was defined as cell in which intranuclear occupancy by the γH2AX signals was less than 1%.

### Clonogenic assays

Clonogenic assays were performed as described previously [28]. Briefly, cells were seeded in 6-well plates and incubated for 12 h to allow cell attachment to the plates. Cells were either exposed to carbon ions or sham irradiated. After incubation for an additional 10 days, cells were fixed with 25% methanol (FUJIFILM Wako Chemicals, Osaka, Japan) and stained with 0.1% crystal violet (Sigma-Aldrich). Colonies comprising at least 50 cells were counted under an inverted microscope. The surviving fraction was calculated by dividing the number of colonies by the number of seeded cells per irradiated sample, which was then divided by the plating efficiency (calculated using unirradiated controls). A cell killing effect was defined as the complementary event of the surviving fraction (i.e. 1 - surviving fraction). All experiments were performed in triplicate.

### Monte Carlo calculation of clinical carbon ion beams

The dose-averaged LET (${L}_{mix}$) at the center of the SOBP was calculated by the particle therapy system simulation framework PTSim [29], which is a software application within the GEANT4 simulation toolkit [30, 31]. The procedure is similar to that described in our previous work [12]. A scatterer made of 2.3 mm-thick lead and a water phantom were defined for the simulation. Generated carbon ions of 290 MeV/n pass through the scatterer and deposit their energy in the water. The distribution of LET and that of the dose, at each depth of water, were recorded in 0.1 mm increments. The *i*-th component of LET distribution at the center of each SOBP (${L}_i$) is calculated as follows:


(1) \begin{equation*} {L}_i={\sum}_j{w}_j{L}_{i, mono}\left(z+{s}_j\right), \end{equation*}

where ${L}_{i, mono}(z)$ is the *i*-th component of monoenergetic LET distribution at depth z, and ${w}_j$ and ${s}_j$ represent the weight and amount of shift for the *j*-th monoenergetic beam component, respectively. The values of ${w}_j$ and ${s}_j$ used in this study are the same as those used to design the ridge filter for each SOBP. ${L}_{mix}$ for the clinical carbon ion beams is determined as follows:


(2) \begin{equation*} {L}_{mix}=\sum{L}_i{d}_i/D, \end{equation*}

where ${L}_i$ and ${d}_i$ is the LET of the *i*-th component and its dose contribution, respectively, and $D$ is the total dose of the mixed LET beam. When calculating ${L}_{mix}$, carbon ions with LET of > 100 keV/μm were recognized as ions with LET = 100 keV/μm, based on the assumption that the lower limit of the overkill region is approximately 100 keV/μm [9–11]; therefore, the death-fate of irradiated cells in the overkill region is immutable.

### Mathematical and statistical analyses

Approximation of the induction probability of γH2AX foci with a normal distribution was performed using ROOT (version 5.34, European Organization for Nuclear Research, Genève, Swiss) by employing the least-square methods with a bin width of 0.1 μm^3^. Approximation of the ratio of unhit cells to the entire irradiated cell population versus LET was performed using Microsoft Excel (version 16, Microsoft, Redmond, WA, USA). Correlations between two factors were examined using Spearman’s rank correlation test in GraphPad Prism 8 (GraphPad Software, San Diego, CA, USA), Statistical significance was set at *p* = 0.05.

## RESULTS

**Fig. 1:**
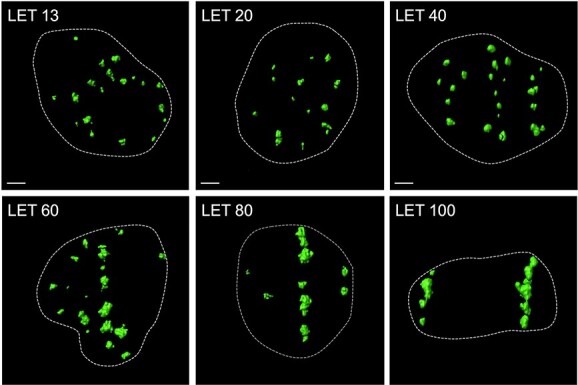
Representative high-resolution images of γH2AX foci induced in A549 cells by monoenergetic carbon ion beams with various LET. Cells were irradiated with 1 Gy carbon ions (LET: 13, 20, 40, 60, 80, or 100 keV/μm), fixed 30 min post-irradiation and stained with γH2AX (green) and DAPI (to identify nuclei; not shown). 3D-deconvolution images of γH2AX foci were obtained using DeltaVision OMX, followed by surface polygon rendering by Imaris 8.1.2. Scale bars, 2 μm. Dashed lines delineate nuclei.

To investigate the relationship between LET and the degree of DSB clustering after exposure to carbon ions, we used the 3D-deconvolution imaging in the DeltaVision OMX system to analyze the volume of γH2AX foci induced by irradiation of monoenergetic carbon ion beams. In this study, we used 1BR hTERT human dermal fibroblasts and A549 human lung adenocarcinoma cells as the representative of normal and tumor cells, respectively. We chose A549 because this line shows a comparable number of DSBs and similar repair kinetics to normal cells after IR [32]. The LET examined ranged from 13 keV/μm to 100 keV/μm based on the fact that the former corresponds to the LET at the entrance region of the clinical carbon ion beams, and the latter is around the highest LET known to be absent for the overkill effect [10]. In cells irradiated with 13–40 keV/μm beams, small granular shaped γH2AX foci were observed predominantly, showing a spatially homogeneous distribution (Fig. 1 and Supplementary Fig. 1). By contrast, in cells irradiated with a beam at 60 keV/μm or greater, γH2AX foci presented huge and complex morphology along the particle track (Fig. 1 and Supplementary Fig. 1). These observations are consistent with our previous study using the 3D-SIM technology to analyze the γH2AX foci induced in 1BR hTERT cells irradiated with 20 keV/μm or 60 keV/μm carbon ion beams [20], suggesting the reliability of the data obtained in the present study.

**Fig. 2:**
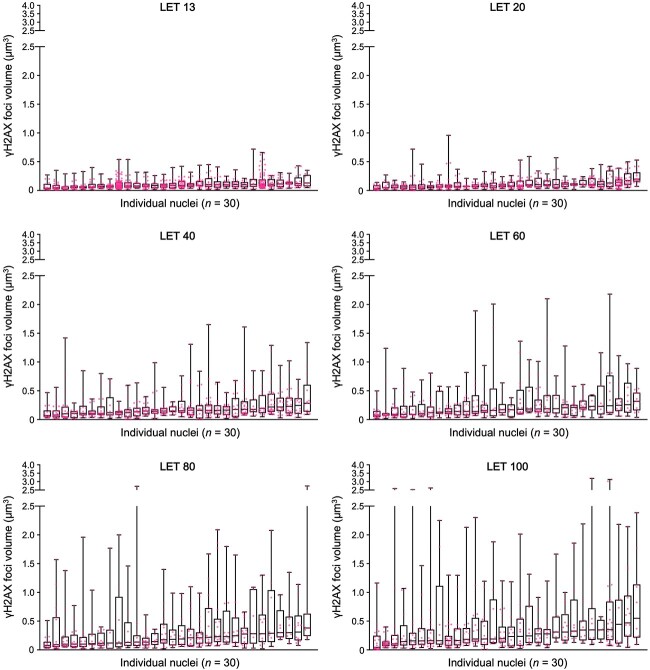
Volume of γH2AX foci induced in individual nuclei of A549 cells (*n* = 30) by monoenergetic carbon ion beams of various LET. Cells were irradiated with 1 Gy carbon ions (LET: 13, 20, 40, 60, 80, or 100 keV/μm), fixed 30 min post-irradiation and stained with γH2AX and DAPI. High-resolution images of γH2AX foci were obtained using DeltaVision OMX, followed by surface polygon rendering by Imaris 8.1.2.

**Fig. 3:**
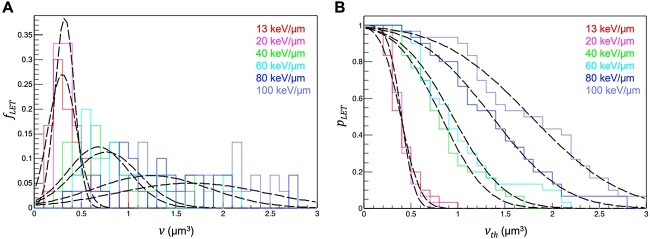
Relationship between the LET of monoenergetic carbon ion beams and the volume of γH2AX foci. (**A**) $f(v)$. Dashed lines show the approximation obtained using formula (3). (**B**) $p$. Dashed lines show the approximation using formula (4). Raw data are derived from Fig. 2.

Evaluation of 30 nuclei per LET resulted in a total of 3317 and 2815 γH2AX foci recorded for A549 and 1BR hTERT cells, respectively. The distribution of γH2AX foci volume in a given nucleus was broadly consistent between A549 and 1BR hTERT cells for all LETs examined (Fig. 2 and Supplementary Fig. 2). For the A549 data set (*n* = 30 for each LET), the distribution of the maximum volume of γH2AX foci induced in a nucleus ($v$) was graphed in Fig. 3A; i.e. the y-axis of this graph (${f}_{LET}$) represents the fraction of cells in which the maximum γH2AX foci volume induced in the nucleus by carbon ions of a given LET was $v$. ${f}_{LET}(v)$ was approximated by a normal distribution as follows:


(3) \begin{equation*} {f}_{LET}(v)=\frac{1}{\sqrt{2\pi }{\sigma}_{LET}}\mathit{\exp}\left(-\frac{{\left(v-{\mu}_{LET}\right)}^2}{2{\sigma_{LET}}^2}\right), \end{equation*}

where ${\mu}_{LET}$ and ${\sigma}_{LET}$ are fitting parameters (which can be approximated from the linear functions of LET), expressed as ${\mu}_{LET}=0.0158 LET+0.126$ and ${\sigma}_{LET}=0.00724 LET+0.0312$, respectively (Supplementary Table 1). Fig. 3B is a reverse cumulative distribution of Fig. 3A; i.e. the y-axis of this graph (${p}_{LET}$) is described as the following equation $(4)$:


(4) \begin{equation*} {p}_{LET}={\int}_{v_{th}}^{\infty }{f}_{LET}(v) dv \end{equation*}

Thus, ${p}_{LET}$ represents the fraction of cells in which the maximum γH2AX foci volume induced in the nucleus by carbon ions of a given LET was ${v}_{th}$ or greater. In other words, ${p}_{LET}$ is the probability that carbon ions of a given LET induce γH2AX foci of the volume ${v}_{th}$ or greater per nucleus.

**Fig. 4:**
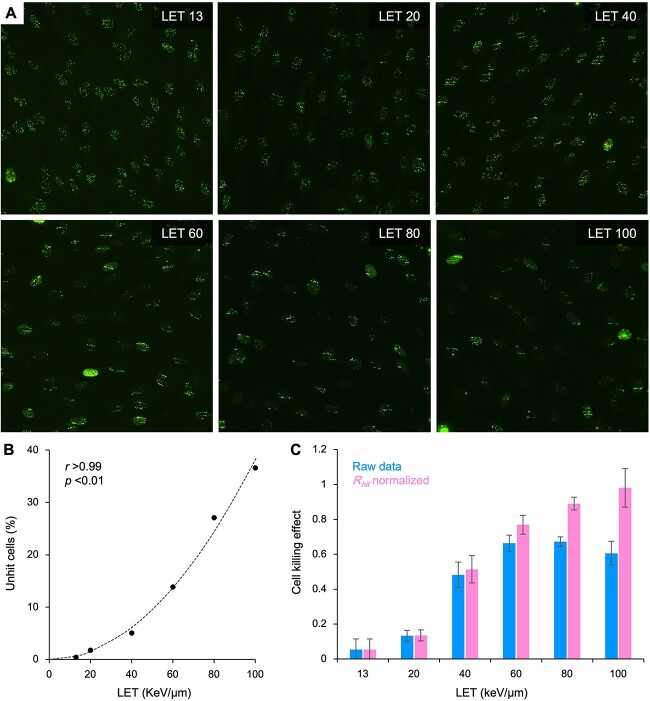
${R}_{hit}$ -normalized cell killing effect of monoenergetic carbon ion beams with various LET. (**A**) Measurement of unhit cell fractions. 1BR hTERT cells, cultured as a confluent monolayer, were irradiated with 1 Gy carbon ions (LET: 13, 20, 40, 60, 80, or 100 keV/μm), fixed 30 min post-irradiation and stained with γH2AX (green) and DAPI (to identify nuclei; see Supplementary Fig. 4). To examine γH2AX foci in irradiated cells with nuclei of similar size, 1BR hTERT cells were synchronized to G0/G1 by contact inhibition. Images of γH2AX foci were obtained using ECLIPSE Ni-U with a DS-Qi2 camera and the NIS-Elements D system software. Representative images are shown for each LET. (**B**) Approximation of the unhit cell fraction. For the images obtained in A, the area of the γH2AX signal, and that of nuclei, was identified using ImageJ. Unhit cells were defined as cells in which intranuclear occupancy of γH2AX signal is less than 1%. At least 200 cells per LET were examined. The plots were approximated using formula (5) and data are shown as a percentage. *r* and *P* values calculated by Spearman’s rank correlation test are shown. (**C**) Cell killing effect (defined as the complementary event of clonogenic survival) of monoenergetic carbon ions at 1 Gy in A549 cells normalized (or not) to ${R}_{hit}$ derived from formula (6) (mean $\pm$ s.d.; *n* = 3).

Upon analysis of the 3D imaging data set, we found that the number of γH2AX foci per nucleus decreased significantly in a LET-dependent manner (Supplementary Fig. 3). This was considered reasonable because higher LET carbon ions cause ionization with greater spatial heterogeneity at the same absorbed dose. In other words, when irradiating with the same absorbed dose, the number of particles passing through a given area within the irradiated target becomes smaller at a higher LET. These data suggest the presence of γH2AX foci-negative (i.e. unhit) cells, especially in higher LET regions. To address this, we used conventional fluorescence microscopy to evaluate γH2AX signal positivity in monolayer-cultured cells irradiated with monoenergetic carbon ion beams with a range of LETs (N.B. we used 1BR hTERT cells for this experiment because 1BR hTERT cells produce better monolayer cultures and show better G0/G1 synchronization to obtain similar nuclear size between analyzed cells). As a result, the fraction of unhit cells increased significantly in an LET-dependent manner, reaching 36.6% at 100 keV/μm (Figs. 4A,4B and Supplementary Fig. 4). Based on these data, the ratio of unhit cells to the entire irradiated cell population versus LET (${R}_{unhit}$) was approximated using a quadratic function as follows:


(5) \begin{equation*} {R}_{unhit}={\left(0.0618 LET\right)^2}/100. \end{equation*}

Based on the formula (5), ${R}_{hit}$ was determined as follows:


(6) \begin{equation*} {R}_{hit}=1-{R}_{unhit}. \end{equation*}

To investigate the relationship between LET and the cell killing effect of carbon ions at 1 Gy, we assessed clonogenic survival of A549 cells irradiated with monoenergetic carbon ions (Fig. 4C). We used A549 cells only for this experiment because the normal fibroblasts 1BRhTERT lack colony forming ability. As expected, the cell killing effect increased in an LET-dependent manner. However, it appeared to plateau at a LET of 60 keV/μm or greater. Interestingly, the plateau disappeared after normalization against ${R}_{hit}$, revealing a strong cell killing effect of carbon ions with a LET of 60 keV/μm or greater. This was considered reasonable because in the clonogenic assays (in which single cells are seeded sparsely and evenly on a culture dish), the surviving fraction after irradiation is composed of the survival of hit-positive cells plus that of hit-negative (i.e. unhit) cells; therefore, the normalization with ${R}_{hit}$ contributes to an accurate estimation of the cell killing effect of carbon ions, especially those with high LET.

**Fig. 5:**
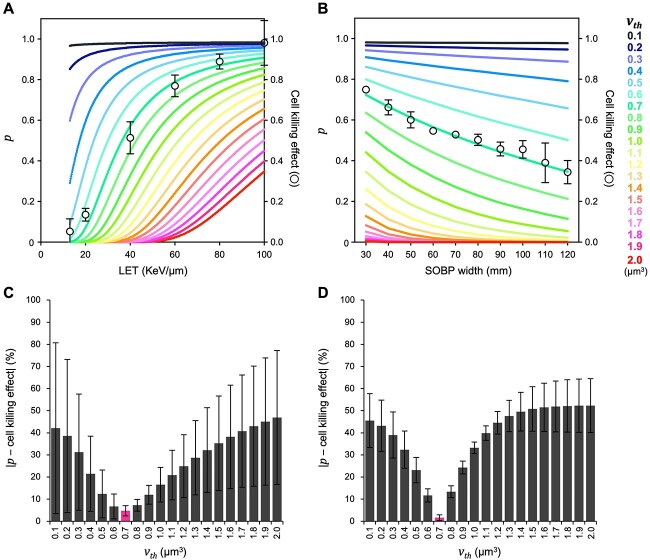
Relationship between the degree of DSB clustering and the cell killing effect of carbon ions. (**A**) $p$ stratified by ${v}_{th}$ (left axis) and the ${R}_{hit}$-normalized cell killing effect (right axis) of monoenergetic carbon ion beams at 1 Gy are shown according to LET. $p$ is calculated based on formula (4). Data used to determine the cell killing effect are derived from Fig. 4C (mean ± s.d.; n = 3). (**B**) $p$ stratified by ${v}_{th}$ (left axis) and the ${R}_{hit}$-normalized cell killing effect (right axis) of clinical carbon ion beams are shown according to SOBP width. $p$ is calculated by substituting ${L}_{mix}$ into formula (4). Data used to determine the cell killing effect were obtained from clonogenic assays at 1 Gy (mean ± s.d.; n = 3). (**C, D**) The difference between $p$ and the ${R}_{hit}$-normalized cell killing effect based on the data shown in panel A and B, respectively.

Based on the data obtained thus far, we next explored the relationship between the degree of DSB clustering and the cell killing effects of monoenergetic carbon ion beams at 1 Gy in A549 cells. When the ${R}_{hit}$-normalized cell killing effect (i.e. a cell killing effect divided by ${R}_{hit}$) was compared with the $p$ for each corresponding LET, the two showed high concordance at ${v}_{th}$ = 0.7 μm^3^ (Fig. 5A and Supplementary Fig. 5). The difference between the ${R}_{hit}$-normalized cell killing effect and $p$ was smallest at ${v}_{th}$ = 0.7 μm^3^ (i.e. 4.7% ± 2.2%) (Fig. 5C). In addition, this phenomenon was also true when the measured values were used to calculate $p$ instead of the formulae (3) and (4), where the difference was smallest at ${v}_{th}$ = 0.7 μm^3^ (i.e. 6.3% ± 5.5%) (Supplementary Fig. 6). These data indicate that the cell killing effect of monoenergetic carbon ions at 1 Gy correlates with the beams to induce γH2AX foci with a specific volume linked to LET.

**Fig. 6:**
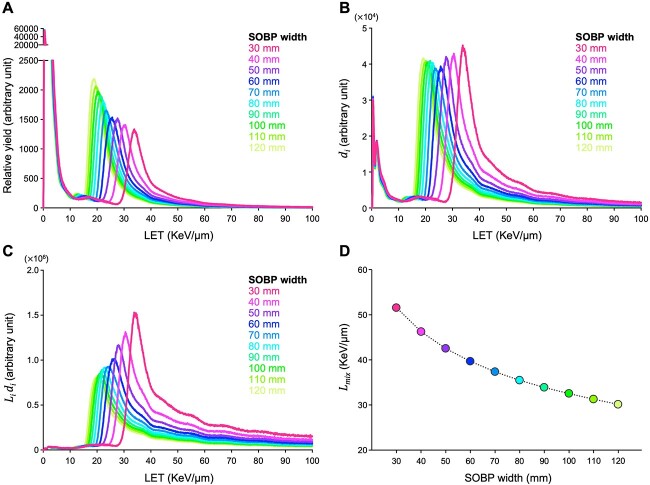
Results of Monte Carlo simulation of clinical carbon ion beams at the center of SOBP. (**A**) Relative yield. (**B**) ${d}_i$. (**C**) ${L}_i{d}_i$. (**D**) ${L}_{mix}$.

Finally, we sought to investigate whether the relationship between LET, the degree of DSB clustering, and the cell killing effect of monoenergetic carbon ion beams at 1 Gy is also true for clinical carbon ion beams that harbor a mixed LET profile. To address this, we performed Monte Carlo simulation to obtain LET profiles at the center of SOBP for clinical carbon ion beams used at GHMC (SOBP width: 30–120 mm in 10 mm increments; see *Materials and Methods* for details). The distribution of carbon ion counts, ${d}_i$ and ${L}_i{d}_i$ (denoting the contribution of carbon ions with each LET to ${L}_{mix}$) against LET is shown in Figs 6A–C. Thus, ${L}_{mix}$ was calculated as shown in Fig. 6D. $p$ at the center of SOBP for the clinical carbon ion beams was calculated by substituting ${L}_{mix}$ into formula (4). The cell killing effect of the corresponding SOBP beams at 1 Gy was examined in clonogenic assays. Here, the cell killing effect was normalized with ${R}_{hit}$ calculated by substituting ${L}_{mix}$ into the equations (5) and (6). As a result, the ${R}_{hit}$-normalized cell killing effect and $p$ at ${v}_{th}$ = 0.7 μm^3^ showed high concordance throughout SOBP width (Fig. 5B); the difference between the two was smallest at ${v}_{th}$ = 0.7 μm^3^ (i.e. 1.6% ± 1.2%) (Fig. 5D). These data suggest that the relationship between LET, the degree of DSB clustering and the cell killing effect of monoenergetic carbon ion beams at 1 Gy can be applied to clinical carbon ion beams harboring mixed a LET profile by employing dose-averaged LET in calculating $p$.

## DISCUSSION

Our analysis of a huge data set of high-resolution 3D γH2AX foci volumes revealed the relationship between the degree of DSB clustering and the cell killing effect of carbon ions for the first time, with LET as the parameter that links the two factors. Data from monoenergetic beams and those from clinical mixed LET beams showed fair agreement when an absorbed dose of 1 Gy was used for experiments. Our data also highlight the importance of considering the unhit fraction when estimating the cell killing effect of carbon ions, especially in high LET regions (i.e. 60–100 keV/μm). In other words, the data raise awareness with respect to mixing the pseudo-reduced cell killing effect mediated by the unhit fraction with the classically recognized overkill effect.

In the clinic, the antitumor effects of CIRT are estimated using the clinical dose unit of Gy (relative biological effectiveness [RBE]), where the RBE values are derived from the clonogenic assays using HSG human salivary gland tumor cells. HSG was chosen as the reference because this cell line shows intermediate radiosensitivity among cell lines of various origins [13]. In the clinic, the optimal dose for each tumor site has been obtained by dose escalation studies. Nevertheless, since the tumor-to-tumor variety of RBE is not included in the definition itself, RBE values vary widely, even among cancer cells originating from the same site or those of the same histopathological type (e.g. the 95% confidence interval of RBE is as wide as 2.1–2.6 for non-small lung cancer cell lines (*n* = 15) [33] and 1.9–2.7 for head and neck squamous cell carcinoma cell lines (*n* = 10)) [34]. These data suggest that calculating the clinical dose using a unified RBE value leads to huge variance in the real antitumor effect of CIRT plans with the same dose prescription. On the other hand, estimation of RBE for individual tumors is highly difficult, even in pre-clinical settings, because RBE is affected by sensitivity to X-rays and to carbon ions, which does not always correlate [26, 33, 34]. We believe that our data provide clues for solving this unmet need from a different perspective; i.e. the relationship between LET, the degree of DSB clustering and cell killing effects. It is worth noting that the A549 cells used in the present study are radioresistant [35]; nevertheless, the degree of DSB clustering was comparable with that observed in 1BR hTERT normal fibroblasts. However, we stress that the magnitude of γH2AX expansion on chromatin can be variable if other doses are used or ATM and its related factors are downregulated. Nevertheless, the induction probability of clustered DSBs according to LET must be universal, irrespective of doses or cell types.

In the future, the relationship between LET, the degree of DSB clustering and the cell killing effect of carbon ions should be investigated under the hypoxic conditions relevant to clinical tumors. Under hypoxic conditions, the ratio of the direct effect to the indirect effect of ionizing radiation changes, and can vary according to LET. Thus, the ratio of the direct/indirect effect, related to oxygen concentration, should be the parameters that affect the relationship between LET, the degree of DSB clustering and the cell killing effect of carbon ions.

There are several limitations in this study. First, as described above, the volume of γH2AX per a break can be changed if other doses are used or ATM and its related factors are downregulated. Indeed, higher doses may generate other type of DSB clustering. However, we believe that the correlation between the induction probability of γH2AX foci and the killing effect of carbon ions will be similarly applied even though the exact value of foci volume is variable depending on other conditions. Second, the exact number of DSBs within γH2AX foci cannot be measured because visualization of individual DSBs is almost technically impossible by immunofluorescence imaging particularly after high LET carbon ion irradiation. Related to this limitation, the detailed mechanism of why 0.7 μm^3^-foci being the threshold of cell killing by carbon ions should be investigated in future. Based on the current understanding, we speculate possible mechanisms below. (i) Two or more DSBs within a γH2AX focus are generated at the boundary of two distinct chromosomes, which cause the formation of lethal chromosome aberrations such as dicentric and translocation. We previously reported that at least > 2–3 DSBs are generated within ~0.7 μm^3^-foci [20]. Since the presence of DSBs was identified by RPA foci, which is a marker of homologous recombination, the number of total DSBs was likely underestimated in the analysis (because DSBs undergoing non-homologous end joining could not be estimated due to the technical difficulty). However, we stress that the distance between DSBs is more important than the number of DSBs within foci. Our previous analysis demonstrated that the average of the distance between two individual DSBs within γH2AX foci was approximately 700 nm [20]. Such close DSBs are rarely introduced after 1 Gy of photons and the formation of 700 nm-two DSBs can form a lethal chromosomal aberration by misrepair because chromatin is compactly folded in a nucleus. Thus, even though the number of DSBs is ~2-3 foci within 0.7 μm^3^-foci, the generation of DSBs in close proximity can be a high risk leading to the formation of lethal chromosomal aberration. (ii) Lethal complexed DNA lesion comprised of DSB, single-strand breaks and base damages within 1–2 helical turns (< 3–4 nm) is high frequently induced within > 0.7 μm^3^-foci. Lastly, in the present study, the 3D volume of γH2AX foci was quantified by high resolution imaging with deconvolution. The image resolution by OMX is significantly greater than other conventional microscopies; however, there is a limit to the resolution of immunofluorescence microscopes. The volume accuracy will be improved by technology advances in future.

The ratio of unhit cells can be calculated from the Poisson distribution with k = 0 with the passing of a particle being the endpoint. Nevertheless, to the best of our knowledge, there is no solid biological evidence on whether one passing of a particle leads to generation of a γH2AX focus. From this perspective, we simply approximated the experimental data with quadratic function not with Poisson distribution.

In conclusion, we demonstrated a high concordance between the induction probability of γH2AX foci of 0.7 μm^3^ or greater and the killing effect of carbon ions (1 Gy) in a given cell. These data may form the biological basis of LET-modulated planning of CIRT.

## Supplementary Material

6_Revised_Supplementary_Materials_221027_rrac098

## Data Availability

Data presented in this manuscript is available upon reasonable request to corresponding author.
