# Supplementary material for: Analysis of the relationship between LET, γH2AX foci volume and cell killing effect of carbon ions using high-resolution imaging technology
Source: J Radiat Res. 2023 Jan 7;64(2):335–44. doi: 10.1093/jrr/rrac098 (PMC10036109; doi:10.1093/jrr/rrac098)
Supplement: 6_Revised_Supplementary_Materials_221027_rrac098 [file 6_revised_supplementary_materials_221027_rrac098.pdf]

Supplementary Table 1. Best-fit values and standard deviation for μ and σ.

LET	μ	σ
13	0.398 ± 0.00403	0.113 ± 0.00591
20	0.39 ± 0.00629	0.164 ± 0.00905
40	0.823 ± 0.01391	0.377 ± 0.0204
60	0.944 ± 0.02277	0.433 ± 0.03512
80	1.362 ± 0.01441	0.604 ± 0.02341
100	1.778 ± 0.02414	0.760 ± 0.03361

Approximation was performed using ROOT (version 5.34) by employing the least-square methods with a bin width of 0.1 μm^3.

Supplementary Fig. 1

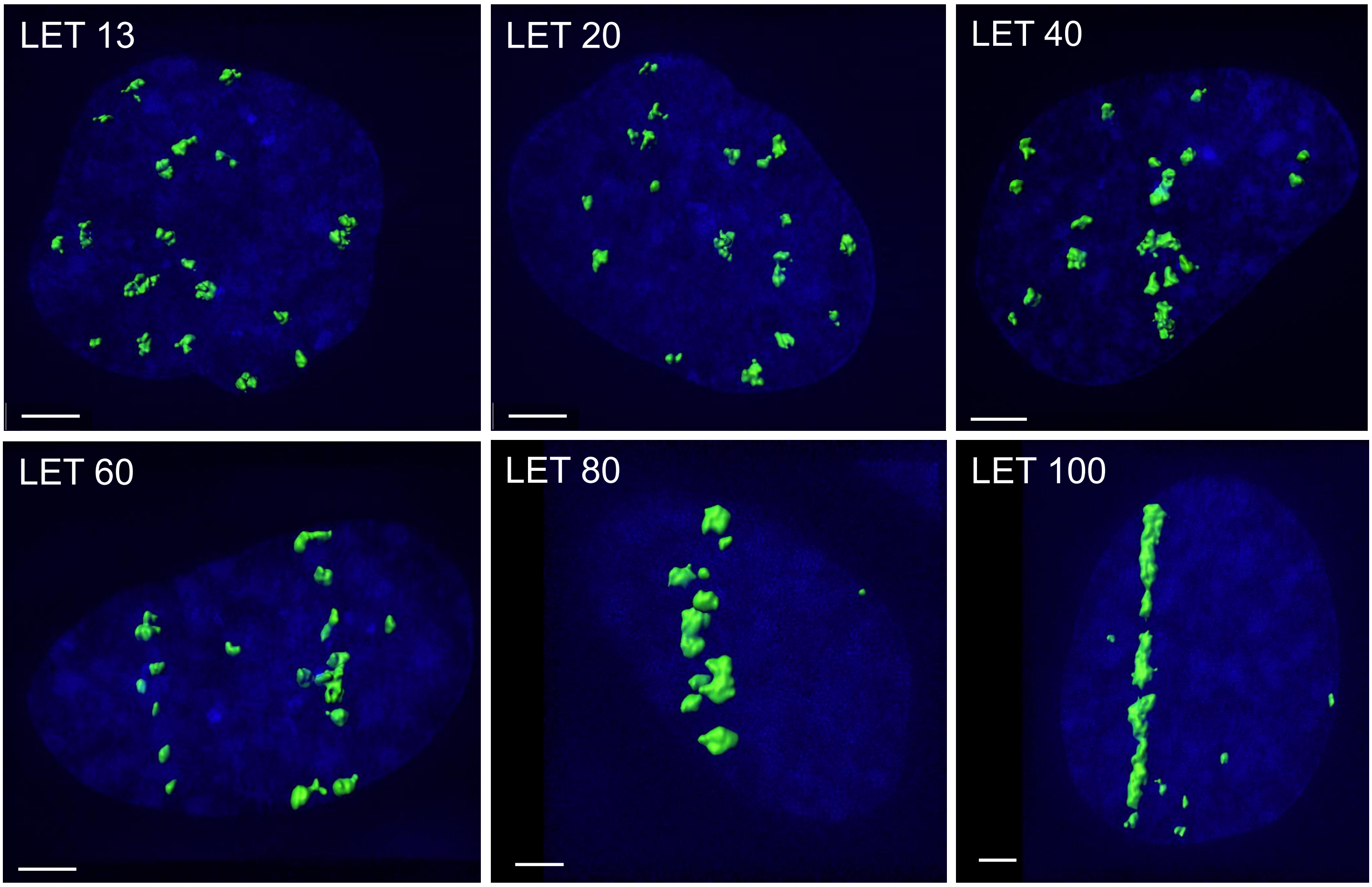

Supplementary Fig. 1. Representative 3D-SIM images of γH2AX foci induced in 1BR hTERT cells by monoenergetic carbon ion beams with various LET. Cells were irradiated with 1 Gy carbon ions (LET: 13, 20, 40, 60, 80, or 100 keV/μm), fixed 30 min post-irradiation, and stained with γH2AX (green) and DAPI (blue). 3D-SIM images of γH2AX foci were obtained using DeltaVision OMX, followed by surface polygon rendering by Imaris 8.1.2. Scale bars, 2 μm.

Supplementary Fig. 2

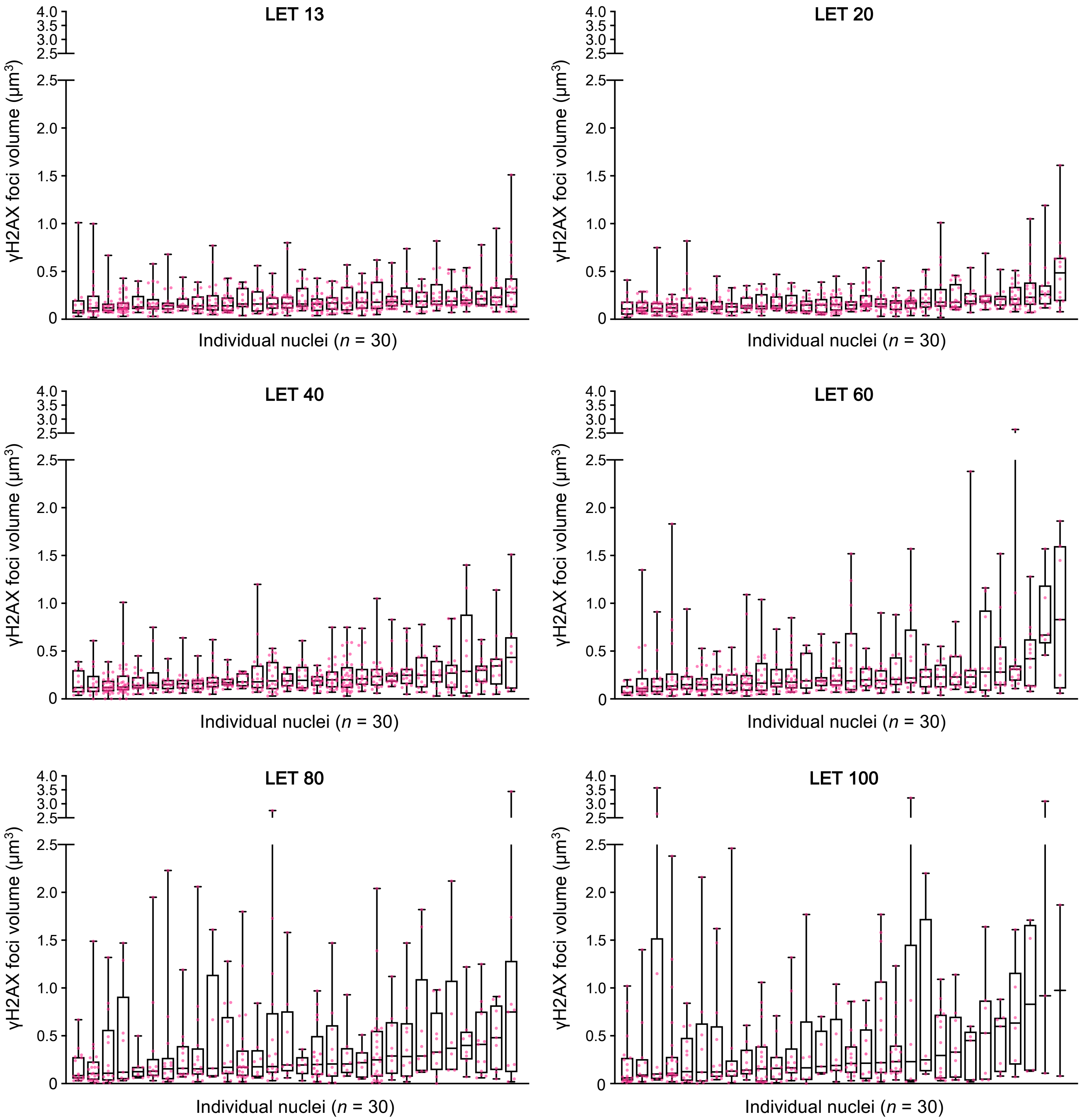

Supplementary Fig. 2. Volume of γH2AX foci induced in individual nuclei of 1BR hTERT cells (*n* = 30) by monoenergetic carbon ion beams with various LET. Cells were irradiated with 1 Gy carbon ions (LET: 13, 20, 40, 60, 80, or 100 keV/μm), fixed 30 min post-irradiation, and stained with γH2AX and DAPI. 3D-SIM images of γH2AX foci were obtained using DeltaVision OMX, followed by surface polygon rendering by Imaris 8.1.2.

Supplementary Fig. 3

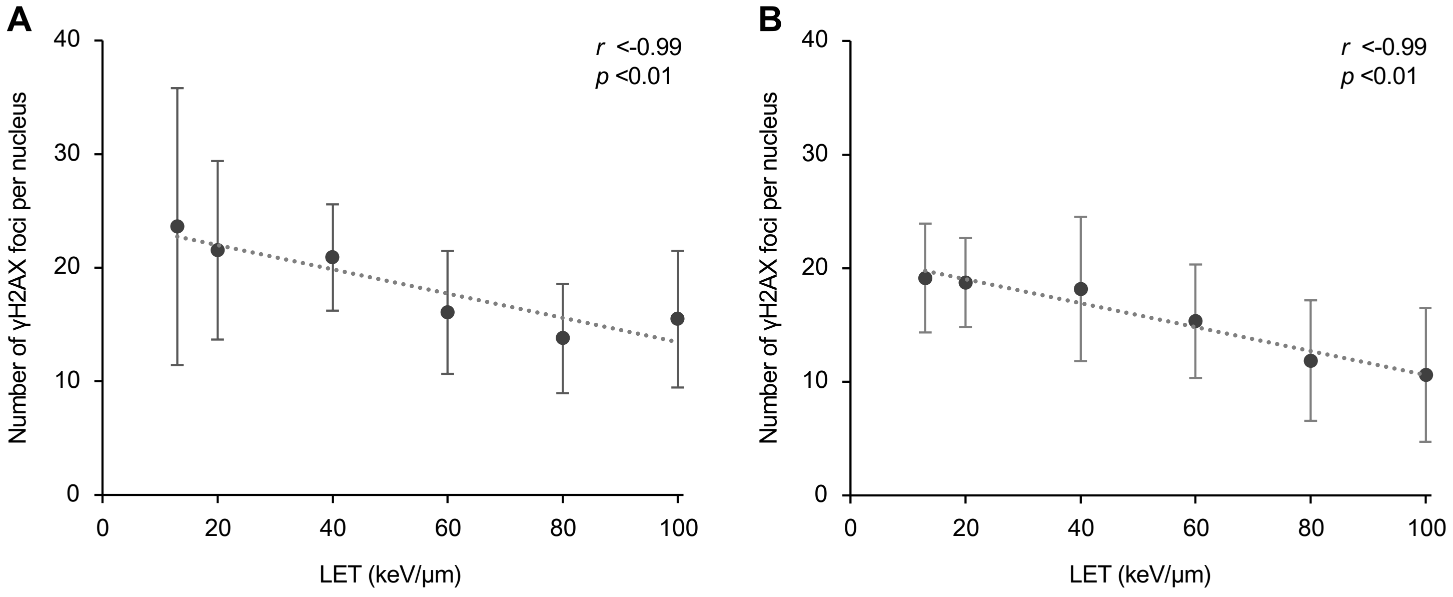

Supplementary Fig. 3. Number of γH2AX foci per nucleus induced by monoenergetic carbon ion beams with various LET. (**A**) A549 cells. Data are derived from Figure 2 (mean ± s.d.; *n* = 30). (**B**) 1BR hTERT cells. Data are derived from Supplementary Figure 2 (mean ± s.d.; *n* = 30). *r* and *p* values, calculated by Spearman's rank correlation test, are shown.

Supplementary Fig. 4

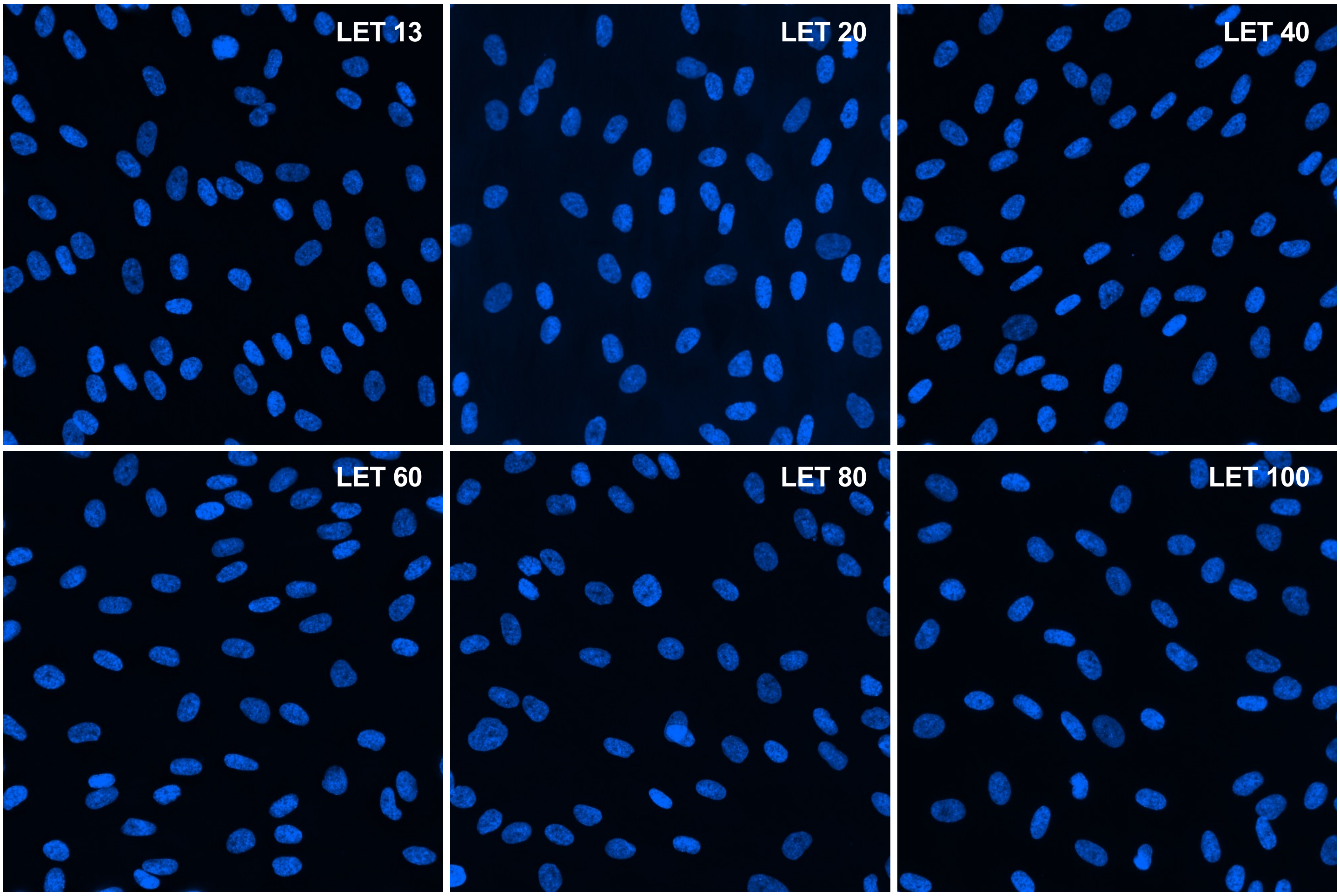

Supplementary Fig. 4. Representative images of DAPI-stained nuclei used to calculate the unhit cell fraction. The samples shown here are the same as those presented in Figure 4A.

Supplementary Fig. 5

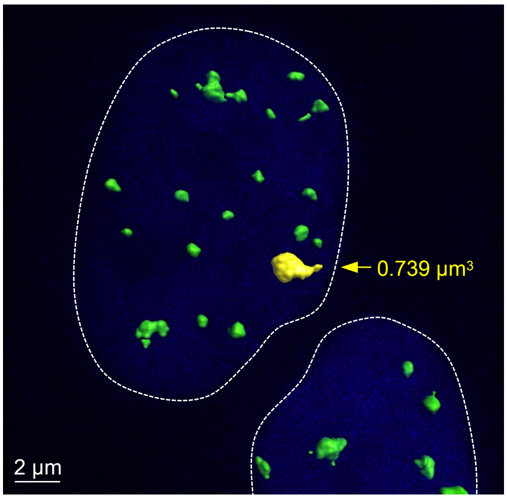

Supplementary Fig. 5. Representative high-resolution images of γH2AX foci with a volume of ~0.7 μm^3 (highlighted in yellow).

Supplementary Fig. 6

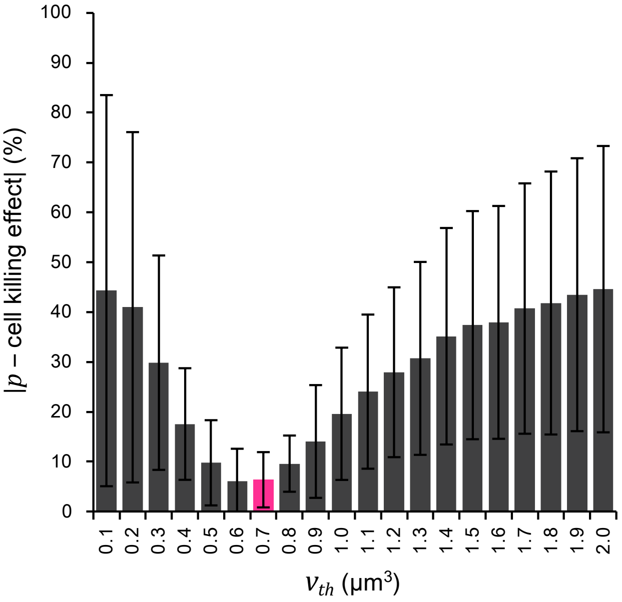

Supplementary Fig. 6. The difference between p and the R_{hit}-normalized cell killing effect in A549 cells irradiated with monoenergetic carbon ion beams for 1 Gy. p is the measured values, not deriving from the formulae (3) and (4).
